# The Effect of NiTi Brush, Polishing Brush, and Chemical Agent on the Dental Implant Surface Morphology and Cytocompatibility

**DOI:** 10.1111/cid.13417

**Published:** 2024-11-21

**Authors:** Giulia Brunello, Kathrin Becker, Nicole Rauch, Frank Schwarz, Jürgen Becker

**Affiliations:** ^1^ Department of Oral Surgery University Hospital of Düsseldorf Düsseldorf Germany; ^2^ Department of Neurosciences, Dentistry Section University of Padova Padua Italy; ^3^ Department of Orthodontics and Dentofacial Orthopedics Charité ‐ Universitätsmedizin Berlin Berlin Germany; ^4^ Department of Oral Surgery and Implantology Goethe University Frankfurt Germany

**Keywords:** hydrophilicity, implant surface modification, implantoplasty, peri‐implantitis

## Abstract

**Objectives:**

To in vitro investigate the effect of different implant surface decontamination methods and treatment storing conditions on implant surface morphology and cell viability.

**Materials and Methods:**

Titanium disks with a sand‐blasted and acid‐etched surface (Promote, *PRO*) were treated with diamond polishing brushes (*BRUSH*), nickel‐titanium brushes (*NITI*), or phenol and sulfuric acid‐gel (*GEL*). The disks were stored in saline (‐S) or left exposed to air overnight (‐A). Untreated (*PRO*) and machined (*MACHINED*) disks were used as controls. *GEL* samples were treated for the 60 s, while the operative time was recorded for *BRUSH* and *NITI*. The samples were subjected to scanning electron microscopy (SEM), surface roughness measurements, and cell viability (SaOS‐2 cells, 7 days) assessment.

**Results:**

The operative time was shorter for *NITI* than for *BRUSH* (*p* = 0.017). The original surface morphology (*PRO*) was not altered in the *GEL* group, in contrast with what was observed for *BRUSH* and *NITI*. The type of storage did not influence the surface morphology. No significant differences in Sa and Sz were observed among the groups, except for MACHINED, which presented lower Sa values (*p* < 0.05). Cells were able to proliferate on all surfaces. *NITI‐S* showed significantly higher cell viability compared to all groups (*p* ≤ 0.001), except for *NITI‐A* and *MACHINED*. Among the treated groups, only one additional significant difference was found, as *NITI‐A* performed better than *GEL‐S*.

**Conclusions:**

None of the investigated protocols compromised the cytocompatibility of the titanium dental implant surface. The best results were registered in the *NITI* group when the samples were stored in saline. Future studies should confirm the effectiveness of the proposed methods in removing bacterial biofilm from contaminated implant surfaces.

## 1 Introduction

Peri‐implantitis has been defined as a plaque‐associated pathological condition affecting peri‐implant tissues, characterized by mucosal inflammation and subsequent progressive bone loss [1, 2]. One of the main goals in peri‐implantitis treatments consists in implant surface decontamination [3]. Thus, owing to its bacterial etiology, the success of the therapy largely relies on the hindrance of the inflammatory process by infection control and effective removal of the biofilm from the surface [4, 5].

Incomplete implant surface decontamination has been advocated as the main responsible factor for unpredictable outcomes of peri‐implantitis treatment. In particular, it is hardly achievable in cases of unfavorable surgical access to the exposed implant threads, as well as in the presence of complex rough surface topography, which is a common feature of the majority of the titanium implants utilized nowadays [4, 6]. Furthermore, the macro‐geometry of the implant could also influence the access of the decontamination devices and their related efficacy [7].

Several decontamination techniques have been proposed, including mechanical, chemical, and physical methods [8, 9, 10, 11, 12, 13]. However, no particular approach has resulted to be clearly superior to the others in the surgical management of peri‐implantitis [3, 4, 6]. Ideally, on one hand, these methods should effectively remove bacterial colonization and re‐establish biocompatibility, and on the other hand, they should not produce any deleterious implant surface modifications, which might impair soft and hard tissue healing as well as favor bacterial recolonization [14].

Surface decontamination can alter the physico‐chemical properties of implant surfaces, thus influencing the peri‐implantitis treatment outcomes. Besides topography and roughness, surface wettability plays a crucial role in osseointegration, with improved results in the presence of hydrophilic surfaces [15, 16].

Starting from the assumption that hydroxylated/hydrated surfaces are unlikely to absorb contaminating hydrocarbons and carbonates from the air [15], after implant decontamination under irrigation, the prolonged contact of the modified implant surfaces with air prior to flap repositioning should be prevented in clinical settings. Similarly, the properties of modified samples could be influenced by the contact with air also in vitro studies, where the samples should not be dried after treatments and should be kept in saline till the examinations.

The present in vitro study aimed to assess the effect of three mechanical or chemical dental implant decontamination methods and two post‐treatment conditions (i.e., stored in saline or left to dry in air) on the cytocompatibility of osteoblast‐like cells seeded onto noncontaminated titanium disks. Complete brushing under physiologic saline preventing any access to air was chosen to simulate the clinical situation where brushed titanium implant surfaces are covered by physiologic saline during treatment and/or blood. The study also aimed at investigating the operative time of the different methods, as well as the morphology and roughness of the modified surfaces.

## 2 Materials and Methods

### 2.1 Titanium Disks

Commercially pure Titanium Grade 4 disks, 5 mm in diameter and 2.5 mm in thickness (CAMLOG Biotechnologies AG, Basel, Switzerland), with two different surfaces were used for the present study: 12 machined disks and 78 disks with sand‐blasted with large‐grit and acid‐etched Promote surface. All the samples were provided sterilized and individually packaged.

### 2.2 Sample Preparation

The disks characterized by Promote surface were randomly assigned to one of the following groups:

1. diamond polishing brushes (A.M. Edelingh Diamond Brushes, Müller & Weygandt GmbH, 63654 Büdingen, Germany) (*BRUSH* group) (*n* = 24);
2. NiTi Brush (Nano, HANS KOREA Co. Ltd., Korea) (*NITI* group) (*n* = 24);
3. Phenole and sulfuric acid‐gel (HybenX gel, Epien Medical, Saint Paul, MN, USA) (*GEL* group) (*n* = 24);
4. Untreated positive control (*PRO* group) (*n* = 14).

The machined samples were used as negative control (*MACHINED* group) (*n* = 14).

All procedures were performed by the same experienced and trained operator (J.B.), inside a laminar flow hood in sterile conditions on a Petri disk.

For *BRUSH* and *NITI* groups, one side of each disk was smoothened using a low‐speed 1:1 contra‐angle handpiece (5000 rpm for *BRUSH* and *NITI* respectively) in immersion in sterile saline, till it presented a uniform visually smooth and shiny surface as in clinical settings. The disks were held with sterile anatomical tweezers and the handpiece was kept at approximately 45° at the treated site (Figure S1). For each disk, a new diamond polishing brush or NiTi brush was used and then replaced. For both the *BRUSH* and *NITI* groups, the operative time was recorded by a second operator with a digital chronometer.

For the *GEL* group, a predetermined operative time of 60 s had been selected in advance, according to the manufacturer's instructions for use. A thin layer of gel was positioned on one side of the disks (Figure S1). After 60 s, the samples were rinsed with sterile saline for 15 s to remove the product.

**FIGURE 1:**
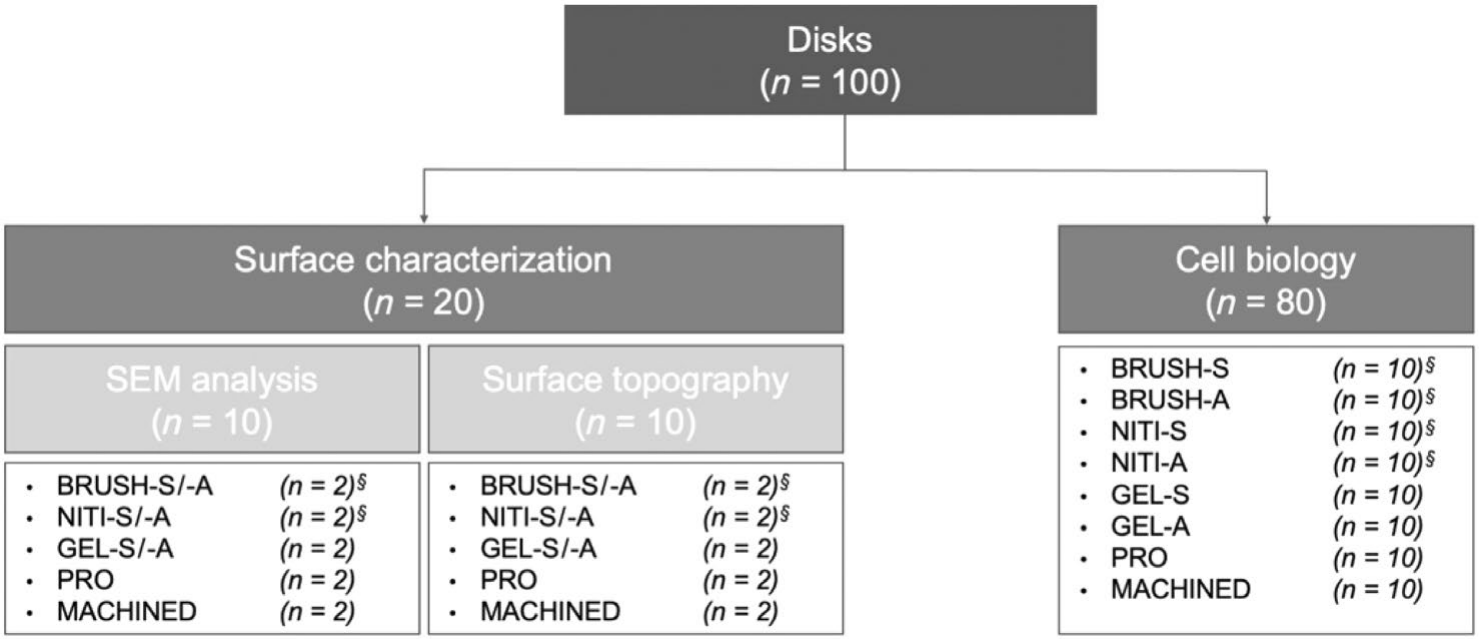
Flowchart of the performed analyses, indicating the number of disks used for each evaluation. ^§^Recorded operative time.

Then the samples were assigned to surface characterization or cytocompatibility evaluation, as illustrated in Figure 1. For surface characterization, the test samples were firstly assessed after storage in saline, dried overnight and then the analyses were performed again on the same samples (‐S/‐A). As regards cytocompatibility, the treated samples were directly stored in sterile saline, thus hindering the adsorption of potential atmospheric contaminants (saline, S), or left to dry in air overnight (air, A). All the treated samples were rinsed with distilled water for 15 s, either immediately before the experiments (S and ‐S/‐A groups) or before drying (A groups).

The control disks (*PRO* and *MACHINED*) were examined only in dry conditions, without rinsing them with saline or distilled water.

### 2.3 Surface Characterization

#### 2.3.1 Scanning Electron Microscopy Analysis

The surface morphology of both test and control disks was investigated by means of scanning electron microscopy (SEM) (Zeiss CrossBeam 550, Zeiss, Oberkochem, Germany). SEM images of treated and control surfaces were taken at different magnifications (i.e., 150×, 750×, 1500×, and 3000×). To avoid chemical changes in surface composition and therefore changed reactivity or protection to modifications, samples were not sputter‐coated with a metal layer as is usually done in the preparation of biological samples for SEM. For this reason, we faced the challenge of charging effects due to nonoptimal conductivity while imaging the surfaces. For the test groups, in order to reduce exposure to air as much as possible, the disks were fixed on the sample holder and left to dry within the vacuum chamber. After the experiment, the sample holder was removed from the chamber maintaining the disks attached in the same position. They were kept in atmospheric conditions overnight and subjected to a second analysis the following day.

For imaging all groups but one (*BRUSH‐S/‐A*), an electron high tension (EHT) of 2.00 kV was chosen. At least two areas per disk were investigated and multiple images at increasing magnification were acquired after zooming in within the identified areas. In the *NITI* and *GEL* groups, images were taken at two different times using the same imaging parameters.

Due to surface damages induced by the electron beam, the scanning protocol had to be modified in the *BRUSH* groups. An EHT = 1.00 kV was adopted and the images before and after exposure to air were not taken from the same areas of the disks.

#### 2.3.2 Surface Topography

The topographical analysis of the disks was performed using the noncontact profilometer cyberSCAN CT 300 (CyberTechnologies GmbH, Ingolstadt, Germany) at a magnification of 800×. The following three‐dimensional surface roughness measurements were used to quantify the surface morphology: Sa (arithmetical mean height) and Sz (maximum height). The region of interest was chosen to avoid manufacturing features that could alter the surface roughness. Each investigated disk consisted of a circular crown presenting a major radius of 2 mm and a minor radius of 1.5 mm with the center of the circular crown corresponding to the center of the disk (Figure S2).

### 2.4 Cell Viability Assay

To investigate the cytocompatibility of the test and control disks, osteoblast‐like cells, that is, SaOS‐2 cells, were utilized (Acc 243, passage 4, German Collection of Microorganisms and Cell Cultures GmbH, Braunschweig, Germany). Cell culture was performed as described in John, Becker, and Schwarz [17]. The disks were positioned into individual wells of a sterile nonbinding 96‐well plate (Costar 9102, Corning, New York, USA), with the treated surface exposed to the medium. Briefly, 5000 SaOS‐2 cells per sample were cultured in 1 mL of Dulbecco's modified Eagle Medium (DMEM high glucose, Glutamax; Sigma‐Aldrich, Merck Group, St. Louis, MO, USA) supplemented with 10% fetal bovine serum (FBS; Sigma‐Aldrich) and 1% penicillin/streptomycin per well. The samples were then incubated at 37°C, 5% CO_2_ concentration, and 95% relative humidity. The culture medium was refreshed every 2 days.

Cell viability was measured on day 7 by means of a luminescence assay (CellTiter‐Glo; Promega, Mannheim, Germany) in a luminometer (Victor X3; Perkin‐Elmer, Rodgau, Germany). In detail, the culture medium was aspirated and the wells containing the disks were gently rinsed with phosphate‐buffered saline (PBS, Sigma‐Aldrich) to wash off the nonadherent cells. Subsequently, the reagent (100 μL) was added and the reading was performed. The signal was measured in counts per second (CPS).

### 2.5 Statistical Analysis

Statistical analysis was performed using the open‐source software R [18]. The sample size was calculated based on a similar study of our group using G*Power (University of Düsseldorf) [19]. Considering a statistical power of 80% and a significance level of 0.05, at least 2 disks per group were required to detect a statistical difference between the groups in terms of cytocompatibility. Experimental data are presented as mean, standard deviation (SD), and median. Comparison between *BRUSH* and *NITI* groups in terms of operative time was performed using the Mann–Whitney *U* test. One‐way ANOVA and post hoc Tukey multiple comparison tests were used to determine the presence of any significant difference in cell viability between the groups. As regards roughness, since measurements were partially paired (in BRUSH, NITI, and GEL groups measurements were conducted on the same disks after different storing conditions) mixed linear models were utilized, followed by multiple comparison tests with Tukey *p*‐value correction method, when indicated. A *p*‐value < 0.05 was considered statistically significant.

## 3 Results

### 3.1 Operative Time

The mean operative time for obtaining a uniform visually smooth and shiny surface was 54.9 s (SD 9.4) and 48.2 s (SD 7.0) for *BRUSH* and *NITI*, respectively (*p* = 0.017). The median time was 54.0 s for the *BRUSH* group and 47.8 for the *NITI*.

### 3.2 Surface Characterization

#### 3.2.1 SEM Analysis

**FIGURE 2:**
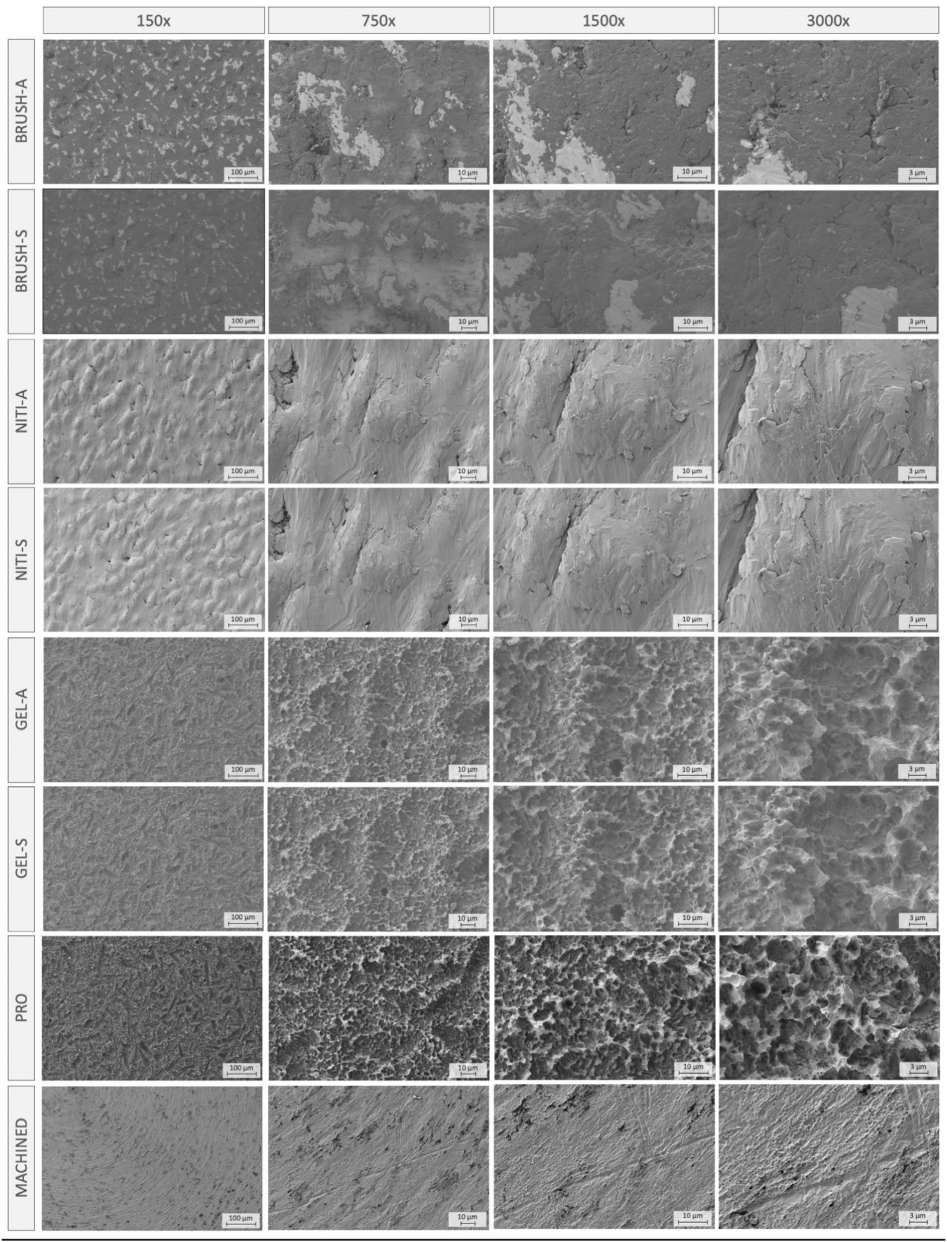
Scanning electron micrographs of treated and controlled titanium disks.

SEM images at different magnifications of treated and control surfaces are shown in Figure 2. Titanium surfaces treated with *GEL* demonstrated the same microstructure as the untreated samples (*PRO*), which were characterized by an irregular surface with peaks alternated with craters and pits. A more pronounced surface modification as compared to *PRO* samples was observed in BRUSH and NITI groups, where the irregular distribution of micro‐pits and sharp edges of the original surface was not detectable. In all test groups, the storage conditions (i.e., saline or air) had no impact on the surface morphology. Machined samples exhibited a relatively flat topography with circumferential marks derived from the production process.

#### 3.2.2 Surface Topography

**FIGURE 3:**
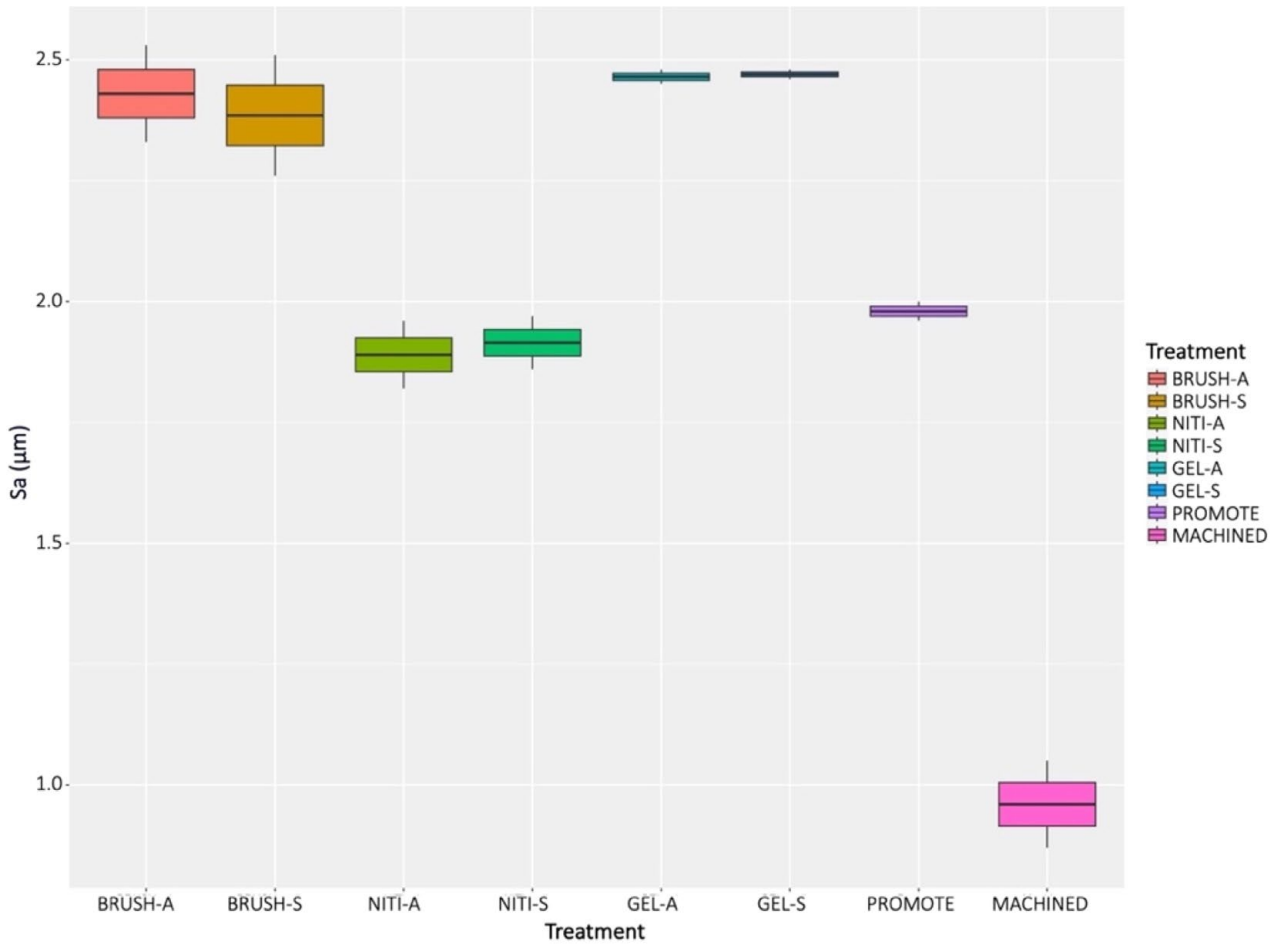
Boxplot showing Sa values of treated and control titanium disks.

**FIGURE 4:**
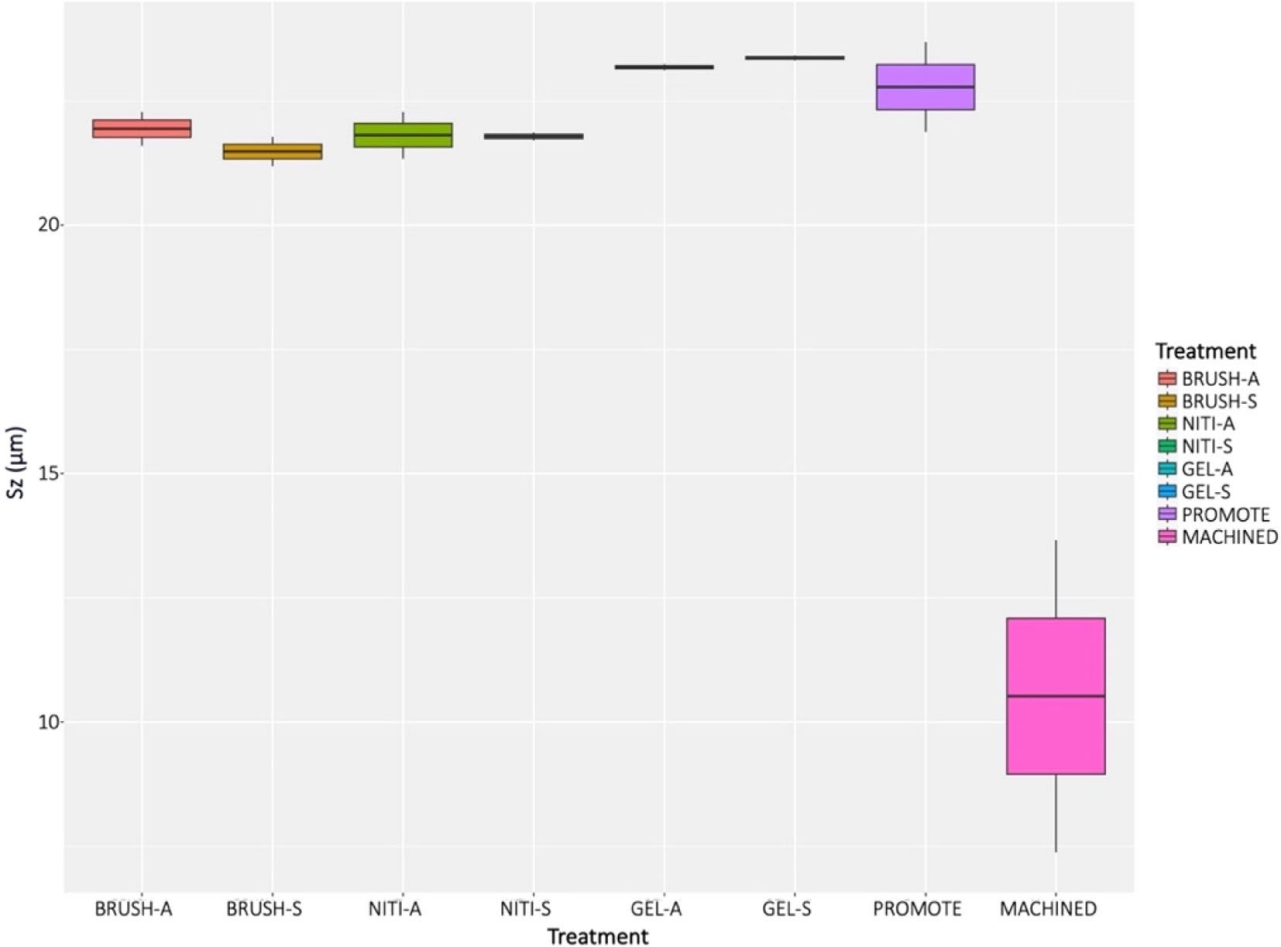
Boxplot showing Sa values of treated and control titanium disks.

3D maps of MACHINED disked demonstrated a different surface texture (Figure S3). This is confirmed by Sa and Sz values which are summarized in Figures 3 and 4, respectively. Significant differences in Sa values were obtained (*p* < 0.001). MACHINED disks resulted in have significantly lower mean Sa value than all the other samples (Table 1), NITI groups showed similar Sa values to the original PRO surface, while an increase in Sa values was observed in the BRUSH and GEL groups, despite not being significant.

**TABLE 1:** Sa and Sz of treated and control disks.

Grouping variable	Comparator	*p* for Sa	*p* for Sz
BRUSH‐A	BRUSH‐S	0.999	1.000
	NITI‐A	0.456	1.000
	NITI‐S	0.519	1.000
	GEL‐A	1.000	1.000
	GEL‐S	1.000	1.000
	PRO	0.685	1.000
	MACHINED	< 0.001***	0.968
BRUSH‐S	NITI‐A	0.571	1.000
	NITI‐S	0.635	1.000
	GEL‐A	1.000	1.000
	GEL‐S	1.000	1.000
	PRO	0.789	1.000
	MACHINED	< 0.001***	0.974
NITI‐A	NITI‐S	1.000	1.000
	GEL‐A	0.372	1.000
	GEL‐S	0.361	1.000
	PRO	1.000	1.000
	MACHINED	0.011*	0.970
NITI‐S	GEL‐A	0.432	1.000
	GEL‐S	0.419	1.000
	PRO	1.000	1.000
	MACHINED	0.008**	0.970
GEL‐A	GEL‐S	1.000	1.000
	PRO	0.595	1.000
	MACHINED	< 0.001***	0.944
GEL‐S	PRO	0.582	1.000
	MACHINED	< 0.001***	0.940
PRO	MACHINED	0.003**	0.953

*Note: p*‐values from the post hoc test are reported. Significant values are labeled as follows: *p < 0.05, **p < 0.01, ***p < 0.001.

The flatter profile of the MACHINED surface was also confirmed by Sz data, whereas all the other surfaces presented a similar rough surface topography with mean Sz values above 20 μm. Significant differences in Sz values were observed among the surfaces (*p* = 0.001). However, no statistically significant difference was registered at the post hoc test, despite what evidenced in the graph (Figure 4). This can be explained by the use of p‐value correction and it is likely that with a higher number of investigated disks the Sz in the MACHINE group would have resulted significant lower as compared to all the other surfaces.

### 3.3 Cell Viability Assay

**FIGURE 5:**
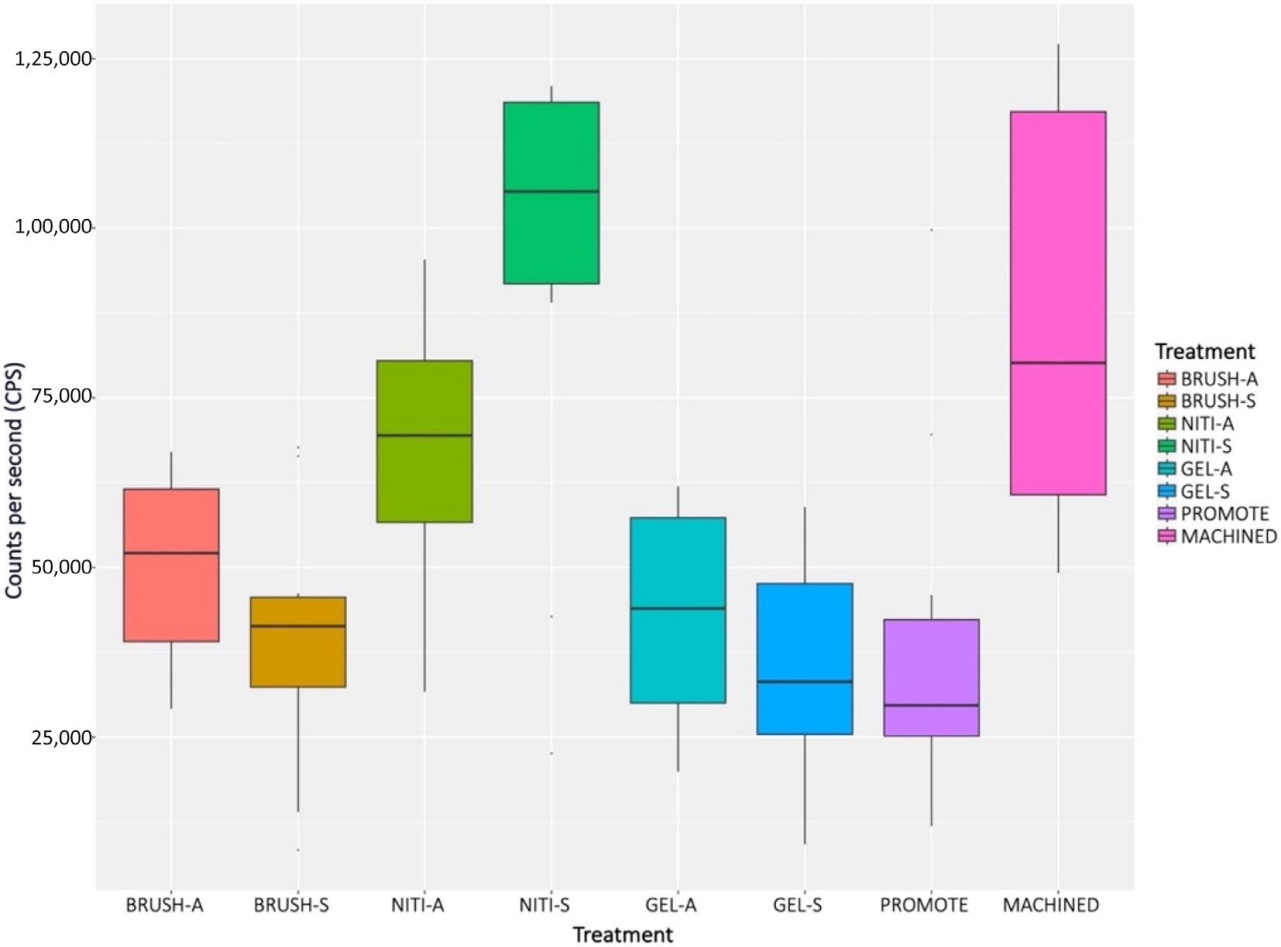
SaOS‐2 cell viability after 7 days of culture on treated and control disks. Data are expressed in counts per second (CPS).

Osteoblast‐like cells were able to proliferate on all treated and control disks, as shown in Figure 5 and Table S1. The highest cell viability values were observed in the NITI‐S group, which presented significantly higher values as compared to all groups, but two (i.e., NITI‐A, MACHINED). Among the other treated groups, no other statistically significant difference was found except for NITI‐A, which exhibited higher values as compared to GEL‐S. Details on the significance level are reported in Table 2.

**TABLE 2:** SaOS‐2 cell viability after 7 days of culture on treated and control disks.

Grouping variable	Comparator	*p*
BRUSH‐A	BRUSH‐S	0.968
	NITI‐A	0.631
	NITI‐S	0.001**
	GEL‐A	0.997
	GEL‐S	0.820
	PRO	0.959
	MACHINED	0.014*
BRUSH‐S	NITI‐A	0.107
	NITI‐S	0.000***
	GEL‐A	1.000
	GEL‐S	1.000
	PRO	1.000
	MACHINED	0.000***
NITI‐A	NITI‐S	0.204
	GEL‐A	0.224
	GEL‐S	0.035*
	PRO	0.095
	MACHINED	0.637
NITI‐S	GEL‐A	0.000***
	GEL‐S	0.000***
	PRO	0.000***
	MACHINED	0.995
GEL‐A	GEL‐S	0.993
	PRO	1.000
	MACHINED	0.002**
GEL‐S	PRO	1.000
	MACHINED	0.000***
PRO	MACHINED	0.000***

*Note: p*‐values from the post hoc test are reported. Significant values are labeled as follows: *p < 0.05, **p < 0.01, ***p < 0.001.

## 4 Discussion

The main aim of this in vitro study was to determine the impact of different implant surface decontamination methods and post‐treatment conditions on the cytocompatibility of osteoblast‐like cells seeded onto noncontaminated titanium disks. Indeed, crucial factors in the management of peri‐implantitis consist in the debridement of the exposed threads and in the concomitant establishment of a surface microstructure able to foster bone regeneration and re‐osseointegration, and to impede, at the same time, bacterial recolonization and the subsequent recurrence of the pathology. The best results in terms of cytocompatibility were obtained with rotating NiTi instruments, in particular when the exposure of the treated surfaces to air was prevented. Debridement with NiTi tools was accompanied by the smoothing of the sharp‐edged microstructure of the PRO surface independently of the storage condition. Therefore, the improved results in the *NITI‐S* group as compared with the *NITI‐A* group might be attributed to the different superficial chemical composition, rather than to its microtexture. Indeed, it is likely that the storage in saline had favored and preserved the formation of a hydroxylated/hydrated surface oxide film [20].

Moderately rough surfaces have been demonstrated to favor the progression of peri‐implantitis to a higher extent as compared to machined implants [12, 21]. To tackle the issues associated with rough implants affected by peri‐implantitis, implantoplasty has been largely applied in combination with both resective and reconstructive approaches. It consists in the removal of the implant threads and of the rough implant surface with the final aim of creating an unfavorable environment for bacterial adhesion and peri‐implantitis recurrence [8, 14]. In surgical nonaugmentative treatment of peri‐implantitis more favorable and stable results have been reported when implantoplasty was performed compared to mechanical debridement alone [22]. Furthermore, a recent systematic review evaluating different surgical modalities for the treatment of peri‐implantitis, confirmed the beneficial effect of implantoplasty applied both in regenerative and nonregenerative surgical approaches on the survival rate and in the resolution of the pathology [23].

It is generally performed with different combinations of rotating instruments in combination or not with finishing Arkansas burs or silicone polishers [24]. Nonetheless, implantoplasty with burs is associated with the reduction of the implant diameter which could lead to the weakening of its structure. Despite it is still controversial whether it has a clinically relevant detrimental effect on the integrity of the implant, caution should be exercised especially when implantoplasty is applied to narrow‐diameter implants [14, 25, 26, 27, 28].

The current in vitro model did not seek to mimic the oral environment in terms of accessibility. However, it is worth mentioning that the presence of adjacent dentition or intraosseous defects commonly impairs the access to the implant surface and could constitute a major issue in the treatment of peri‐implantitis. Two main peri‐implant defect types can be distinguished: intraosseous (Class I) and supracrestal (Class II) defects [29]. While the supracrestal aspect of the exposed implant portion is usually accessible, the configuration of the intrabony component could limit the access of the instruments to the implant surface. This could be further hindered by the presence of a suprastructure [3]. In this context, the recourse to more flexible and conservative tools such as rotating titanium brushes could be particularly advantageous [30, 31, 32, 33]. Titanium brushes may be beneficial in reducing signs of peri‐implant inflammation, despite this cannot be definitive confirmed in light of the current available evidence [10].

In a recent in vitro study, four mechanical decontamination approaches (i.e., ultrasonic devices with stainless steel or PEEK‐coated tips, titanium brushes, and air‐polishing devices) were evaluated using three defect configuration models (types Ib, Ic, and Ie) [34]. The titanium brush proved to be the most effective instrument in the presence of defect walls, in terms of ink removal from the implant surface. In another in vitro study in which a hydroxyapatite coating was regarded as a simulation of contaminated calculi [35], the titanium brush reached the most apical part of the exposed implant surface in the presence of a 3‐mm‐depth circumferential defect (class Ie). However, the effectiveness of the brush declined proceeding toward the apical part of the defect. It is worth noting that in the work of Munakata et al. [35] the morphology of the brush was similar to the one used in the current study, and its efficacy could depend on the inclination of the tip with respect to the implant surface.

Ichioka et al. [36] performed implant surface treatment with NiTi brushes produced by the same company as our instruments, applying slightly different working operative settings, such as a rotational speed of 1200 rpm and the use of threaded implants. They reported a high cleaning efficacy of these instruments, as confirmed by the reduction of a multispecies biofilm from threaded implant surfaces. Both air‐polishing and the NiTi brushes presented not only superior biofilm removal capability as compared to wiping with gauze but also reduced atomic% of Carbon on implant surfaces, which might be associated with enhanced surface hydrophilicity. The unsatisfactory biofilm decontamination obtained from wiping with gauze together with the presence of occasional gauze remnants on implant surfaces might be responsible for the lower cytocompatibility of the so‐treated titanium surfaces as compared to surfaces instrumented with air‐polishing or NiTi brushes [37].

An effective decontaminant effect on previously biofilm‐contaminated implants was demonstrated using the same gel (i.e., HybenX gel) utilized in the present study both alone and in combination with air powder abrasion [38]. Furthermore, the decontamination was followed by a relevant adhesion and growth of MG‐63 osteoblast‐like cells on the treated surface, especially in case of a combined chemical and mechanical approach. As in the present study, despite not being detrimental, the gel‐induced modifications did not lead to an improvement in cellular regrowth [38]. Based on the available literature, despite its good biofilm‐disrupting potential and antimicrobial properties, it seems that this gel cannot substitute mechanical instrumentation [39].

Some inconsistencies between roughness outcomes and the findings by SEM have to be mentioned. All treated disks presented similar roughness values to the original *PRO* surface, regardless of the storage method. Only the *MACHINED* surface exhibited roughness values dissimilar from all the others. However, at SEM analysis the disks treated with *NITI* as well as with *BRUSH* showed a completely altered morphology and the pattern of the original *PRO* surface could not be recognized. Kim et al. [40] investigated the effect of the same NiTi brushes used in the current study on a similar sand‐blasted/acid‐etched titanium surface (SLA), as well as on resorbable blasting media titanium disks. Although it is hard to compare their protocol with ours, as a different speed was utilized (800 rpm) and no indication about the treatment time was provided, a similar surface morphology was also noticed in SEM analysis. Moreover, as regards the roughness, they found significantly lower Ra values after treatment with NiTi brushes than in the other groups (i.e., no treatment; tetracycline; air polisher with glycine powder; and copper alloy ultrasonic scaler tip). On SLA surfaces, the treatment with NiTi brushes was also accompanied by a reduced adherence of *S. gordonii* [40]. By contrast, in another in vitro study the treatment with another type of titanium brush did not result in any significant changes in the three‐dimensional roughness parameters for both machined and sand‐blasted/acid‐etched titanium disks [41].

The flattening of the characteristic sharp peaks of the sand‐blasted/acid‐etched Ti surface was also observed in a previous study from our group [31]. Ti brushes, different from the ones here utilized, were revealed to be more effective than steel curettes in plaque removal after 48 h of in situ plaque accumulation. Moreover, the treated disks were autoclaved and seeded with SAOS‐2 cells to assess their cytocompatibility. Contrary to the current work, the original moderately rough surface presented significantly higher cell viability values after 3 days of culture as compared to both the test groups.

The positive influence of implant surface treatment observed in the *NITI‐S* group on fibroblast growth could be also due to a modification of the superficial elemental composition obtained with the mechanical method and maintained by the storage in saline. The mechanical decontamination and the prevention of contaminations that occurs during air contact might have increased the wettability of the surface [42]. In an in vitro study of Rupp et al. investigating several commercially available implant systems [43], the SLActive surface exhibited the best results in terms of hydrophilicity. Since it showed superior properties also as compared to the SLA surface, which is produced with the same sandblasting and etching procedure, it comes clear that the conservation method played a major role in preserving the hydrophilicity of the surface. Indeed, contrary to SLA implants, SLActive ones are rinsed under nitrogen protection and directly stored in an isotonic solution, again protected by nitrogen filling, in order to avoid air contact [42]. This treatment contributes to the limited amount of absorbed hydrocarbon contaminants, which is deemed to be responsible for the reduced hydrophilicity of titanium implant surfaces [42]. Surface hydrophilicity has been demonstrated to foster bone formation and to exert a pro‐osteogenic and pro‐angiogenic influence on gene expression [44, 45, 46], and it would be highly recommended especially in combination with regenerative techniques in the context of peri‐implant treatment. However, a drawback of this study was the lack of investigation of the wettability of the treated surfaces via contact angle measurements to confirm this hypothesis.

The absence of microbiological tests confirming the efficacy of the proposed protocols to disrupt bacterial biofilm from contaminated surfaces represents the major shortcoming of the current investigation. Future developments of this study should be addressed to explore the effectiveness of the tested decontamination methods in terms of biofilm removal. Furthermore, the present study was confined to a laboratory setting, without the common challenges that the clinicians have to face, including variable difficult accessibility in relation to the location of the implant or the morphology of the defect, the macro‐geometry of threaded implants as well as the presence of prosthetic supra‐structures. Therefore, the results of these investigations should be cautiously extrapolated to clinical scenarios and the advantages of the *NITI* protocol should be confirmed in controlled clinical trials. Finally, it would be interesting to verify whether treatment with NiTi brushes without subsequent exposure to air could lead to the establishment of hydrophilic or ultrahydrophilic implant surfaces.

## 5 Conclusions

In conclusion, within the limitations of this in vitro study, all tested implant surface treatments on noncontaminated titanium disks could be considered valid approaches in terms of cytocompatibility. The treatment with gel and polishing brush demonstrated comparable cytocompatibility compared to the original promote surface. Implantoplasty involving the removal of the superficial promote surface layer with NiTi brushes without allowing exposure to air demonstrated significantly better biocompatibility, while contact with air reduced the biocompatibility in the NiTi brush group.

## Author Contributions

J.B. conceived the ideas; G.B. and N.R. collected the data; G.B. and K.B. analyzed the data; G.B. and J.B. obtained the funding; F.S. and J.B. supervised the project; G.B. and K.B. led the writing. All authors critically revised the manuscript and agreed to the final version.

## Conflicts of Interest

The authors declare no conflicts of interest.

## Supporting information


**Data S1.** Supporting Information.

## Data Availability

The data that support the findings of this study are available from the corresponding author upon reasonable request.
