# Supplementary material for: The Effect of NiTi Brush, Polishing Brush, and Chemical Agent on the Dental Implant Surface Morphology and Cytocompatibility
Source: Clin Implant Dent Relat Res. 2024 Nov 21;27(1):e13417. doi: 10.1111/cid.13417 (PMC11798888; doi:10.1111/cid.13417)
Supplement: Supplementary file 1 — Data S1. Supporting Information. [file CID-27-0-s001.docx]

***
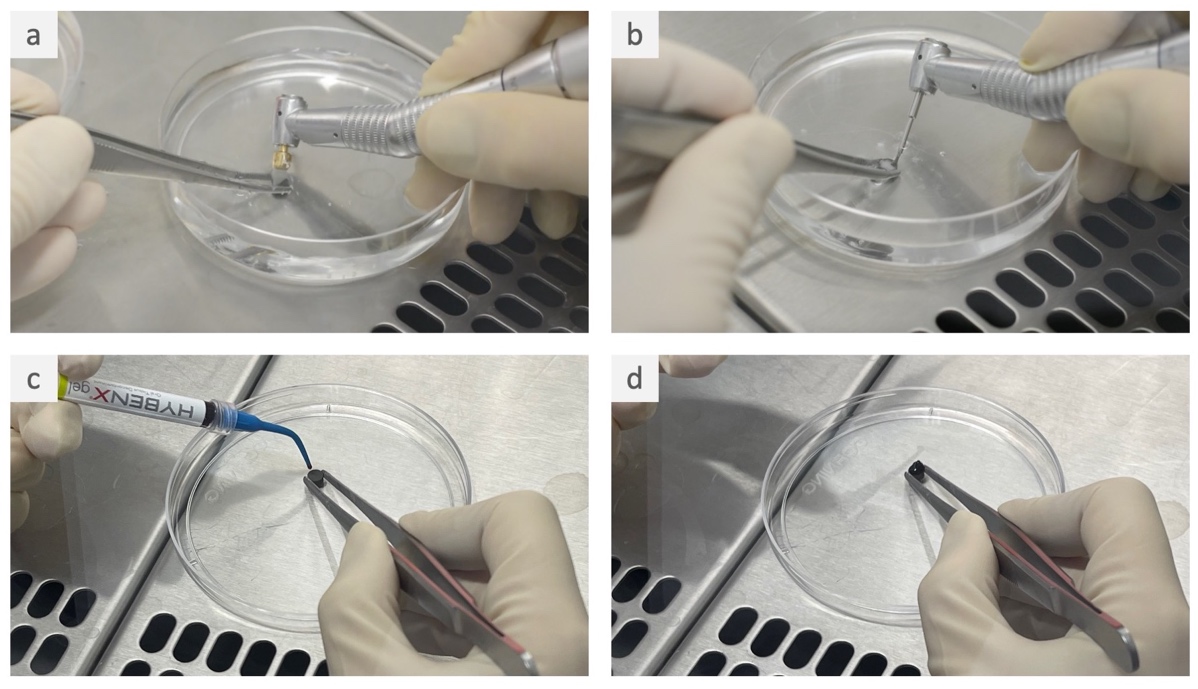
***

***Figure S1.*** *Ti disk surface modifications: a) using a diamond polishing brush, BRUSH; b) utilizing a NiTi brush, NITI; c,d) by applying a chemical agent, GEL.*


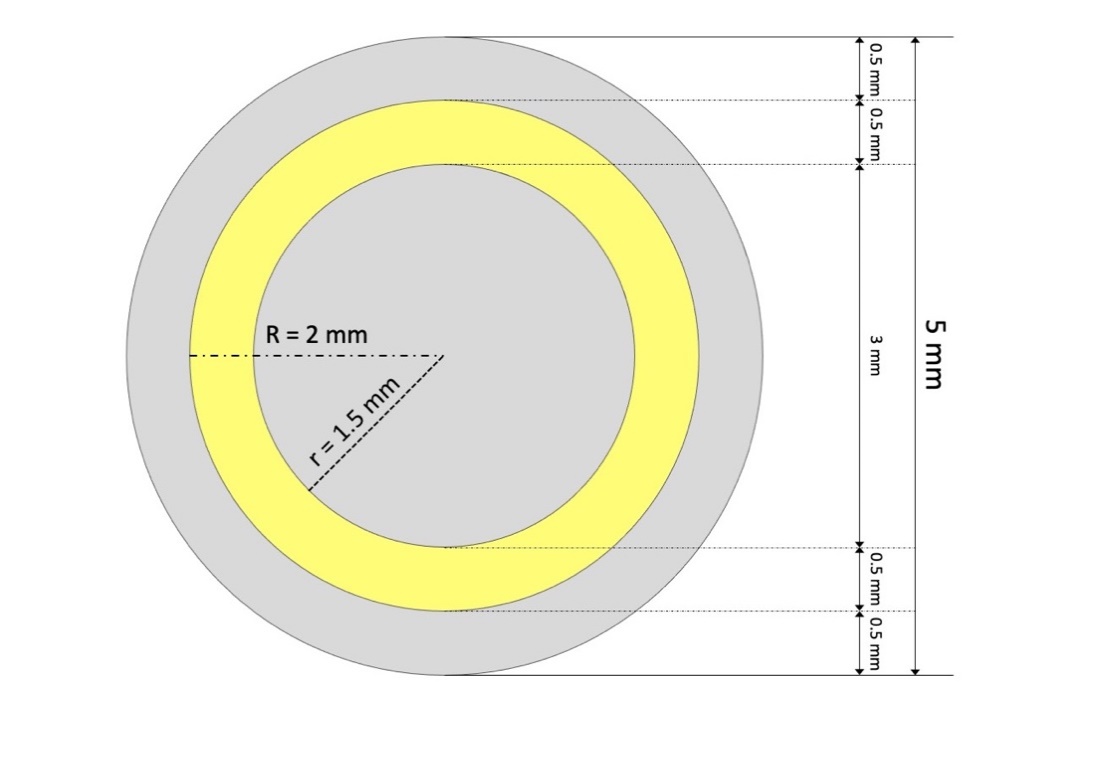


***Figure S2.*** *Region of interest for surface topography measurements (in yellow) of control and test disks (grey). The major radius and the minor radius of the circular crown utilized for the measurements are indicated with “R” and “r”, respectively.*

***
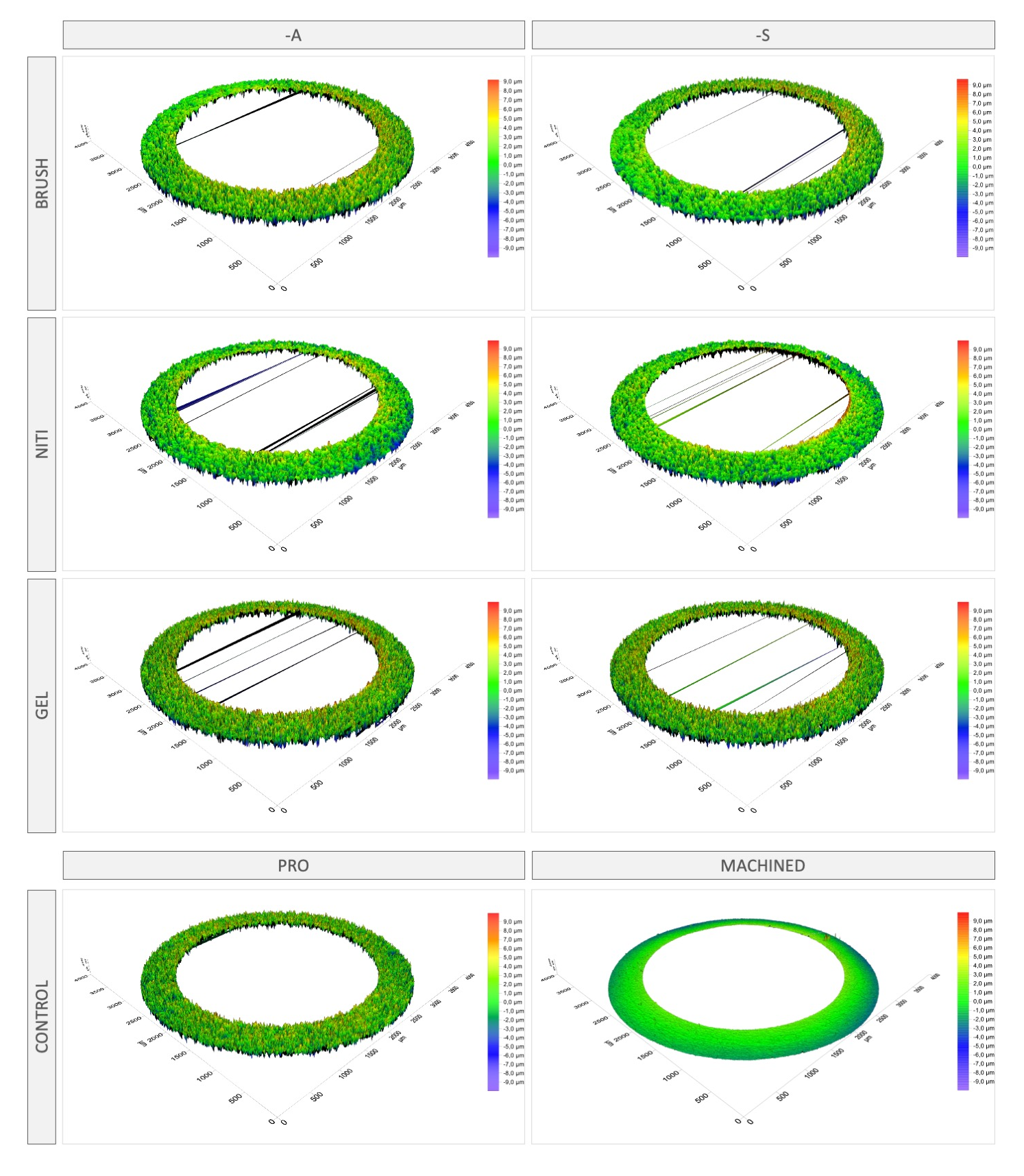
Figure S3.*** *3D maps of treated and control titanium disks.*

**Table S1**. SaOS-2 cell viability after 7 days of culture on treated and control disks. Data are expressed in counts per second (CPS).

| **VALUE** | **BRUSH-A** | **BRUSH-S** | **NITI-A** | **NITI-S** | **GEL-A** | **GEL-S** | **PRO** | **MACHINED** |
| --- | --- | --- | --- | --- | --- | --- | --- | --- |
| MEAN | 50362.7 | 39842.8 | 68599.6 | 94310.2 | 43364.6 | 35336.9 | 39313.9 | 86736.8 |
| SD | 13510.2 | 19140.2 | 19251.5 | 34429.8 | 15639.9 | 15283.7 | 26335.1 | 29859.7 |
| MEDIAN | 52151 | 41384 | 69457.5 | 105391.5 | 43982 | 33188 | 29687 | 80158 |
